# Assessment of sample freezing as a preservation technique for analysing the molecular composition of dissolved organic matter in aquatic systems†

**DOI:** 10.1039/d3ra01349a

**Published:** 2023-08-16

**Authors:** Jeremy A. Fonvielle, Stacey L. Felgate, Andrew J. Tanentzap, Jeffrey A. Hawkes

**Affiliations:** a Ecosystems and Global Change Group, Department of Plant Sciences, University of Cambridge Cambridge CB2 3EA UK; b Department of Chemistry – BMC, Uppsala University Husargatan 3 Uppsala 752 37 Sweden jeffrey.hawkes@kemi.uu.se; c Ecosystems and Global Change Group, School of the Environment, Trent University Peterborough K9L 0G2 Canada

## Abstract

Dissolved organic matter (DOM) is widely studied in environmental and biogeochemical sciences, but is susceptible to chemical and biological degradation during sample transport and storage. Samples taken in remote regions, aboard ships, or in large numbers need to be preserved for later analysis without changing DOM composition. Here we compare high-resolution mass spectra of solid phase extractable DOM before and after freezing at −20 °C. We found that freezing increases compositional dissimilarity in DOM by between 0 to 18.2% (median = 2.7% across 7 sites) when comparing replicates that were frozen *versus* unfrozen, *i.e.*, processed immediately after sampling, as compared with differences between unfrozen replicates. The effects of freezing primarily consisted of a poorer detection limit, but were smaller than other sample preparation and analysis steps, such as solid phase extraction and variable ionisation efficiency. Freezing samples for either 21 or 95 days led to similar and only slight changes in DOM composition, albeit with more variation for the latter. Therefore, we conclude that sample freezing on these time scales should not impede scientific study of aquatic DOM and can be used where it makes logistical sense, such as for large spatial surveys or study of archived samples.

## Abstract 2

We assess the effect of sample freezing on the data collected from dissolved organic matter samples by high resolution mass spectrometry, and find that this preservation technique is appropriate if necessary.

## Graphical abstract

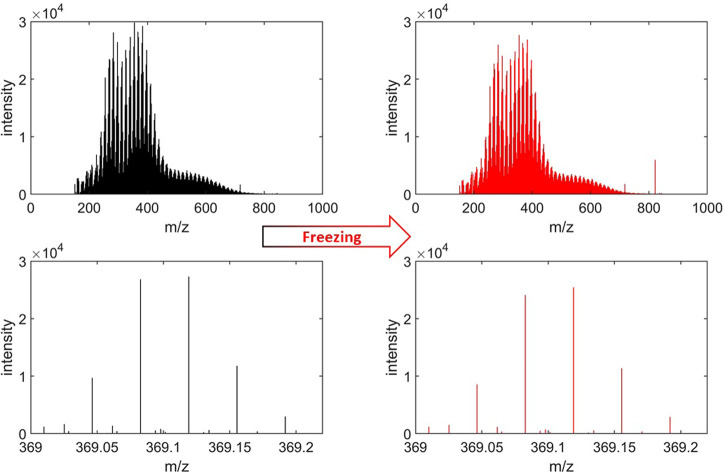

## 1. Introduction

Characterising dissolved organic matter (DOM) composition is critical in environmental and biogeochemical studies, but technical and logistical limitations often restrict the number of samples that can be immediately processed.^1,2^ For instance, sampling sites can be far from laboratories,^3^ and intensive sampling campaigns are often condensed over a short period with limited time for sample preparation.^4^ As additional constraints, ultra-high-resolution mass spectrometry (UHRMS), such as Orbitrap or Fourier-transform ion cyclotron mass spectrometry, which is the gold standard method to measure DOM composition,^5^ often requires a time-consuming solid phase extraction (SPE) step that is traditionally performed as soon as possible after sampling to avoid sample degradation.^6^ Therefore, without a way to preserve collected samples, only a limited number can be analysed, which limits our understanding of global biogeochemical cycles. To allow DOM characterisation in remote areas, on archived samples, and to embrace the realisation of high throughput environmental studies, long-term storage solutions are needed.

Sample poisoning and freezing are among the most used techniques to preserve DOM and prevent biological and photochemical degradation,^7^ but no studies have tested how they affect DOM composition measured using UHRMS. Adding inorganic chemicals (*e.g.* HgCl_2_, NaCl) into samples is cost-efficient and can be performed anywhere under any conditions.^7^ However, most substances that prevent biological activity are harmful to humans and the environment,^8^ and cause difficulties for sample transport because of hazardous goods regulations. In the case of nontoxic chemicals (*e.g.* NaCl), downstream complications arise such as the requirement of several washing steps to remove the excess of inorganic salts prior to processing samples with UHRMS.^6^ Moreover, introducing a compound inside a sample increases the likelihood of sample modification or contamination. Preserving samples at sub-zero temperatures is a simple and cost-effective alternative that is achievable in all areas with electricity. Once frozen, samples can be stored for months without further effort. There are also many laboratories with decades of archived frozen samples that might be appropriate for compositional analysis using modern techniques.

Evidence from studies measuring dissolved organic carbon (DOC) in both marine and freshwater samples after freezing are encouraging, with DOC concentrations remaining unchanged for several months after initial freezing.^9–11^ Although freezing samples changes DOM composition measured using optical properties,^9,11,12^ these effects may not be relevant to UHRMS. A major drawback of freezing water is the formation of crystals which alter the three-dimensional (3D) structure of molecules.^13^ Differences in 3D structure can modify DOM optical properties,^14^ but 3D structures are not resolved in UHRMS. While freezing may also modify DOM by rupturing microbial cells and releasing intracellular compounds, these cells are typically removed during pre-filtering of samples through 0.20, 0.45, or 0.70 μm, which are operational definitions for DOM.^6^ Therefore, it is likely that the molecular formulae in DOM are conserved after freezing, even though the 3D configurations have been altered. Overall, the inconveniences of sample poisoning likely outweigh their benefits, whereas freezing samples may be more suitable for preserving samples before UHRMS measurements.

The initial DOM composition of a sample might further affect its resilience to freezing. Natural DOM comprises diverse compound groups with various structures, ranging from large aromatic (*e.g.* lignin, tannins) to small aliphatic (*e.g.* lipids, peptides)^15^ compounds. Solely based on chemical processes, molecular formulas that are more reactive may be more affected by freezing than compounds decaying at slower rates.^16^ Conversely, compounds that are more prone to flocculation may be more susceptible to loss during freezing. Therefore, environments dominated by highly unsaturated and phenolic compounds, such as boreal lakes,^16^ might be affected differently by freezing than environments containing higher proportions of aliphatic compounds, such as small clearwater streams,^17^ marine waters,^18^ or aerosols.^19,20^ Understanding how compositional responses to freezing vary among sample types is therefore essential to guide preservation efforts.

In this study, we studied the effects of freezing on UHRMS DOM composition (mass spectra and common peak metrics) in two separate experiments. The first experiment investigated fresh and coastal marine waters in Sweden before and after 21 days of storage at −20 °C following a standard protocol for marine studies.^21^ In the second experiment, we investigated freshwaters in the UK during a longer freezing period (95 days) following a protocol designed for a global scale study. We only tested the effect of freezing at −20 °C, as it is the typical temperature used in previous studies.^9,11^ Our results indicate that freezing had a very minor effect on UHRMS results, and so we propose freezing as a sensible option to preserve samples where necessary.

## 2. Materials and methods

### 2.1 Reagents

LC-MS grade methanol (Supelco LiChroSolv hypergrade) and fuming hydrochloric acid (37% HCl) were obtained from Merck Life Science AB, UK. Acetonitrile (ACN) was obtained from Sigma-Aldrich (Supelco LiChroSolv hypergrade for LC-MS) and formic acid (FA) was obtained from VWR (AmalaR Normapur, VWR Sweden). Suwannee River natural organic matter (SRNOM) was obtained from the International Humic Substances Society (IHSS, Saint-Paul, USA; batch number 2R101N). Hippuric acid, fusidic acid, cyclohexyl succinic acid, capsaicin, adenosine-5-monophosphate, raffinose, carbenoxolone disodium and glycyrrhizic acid were obtained from Merck Life Science AB, Sweden.

### 2.2 Experimental design

**Fig. 1:**
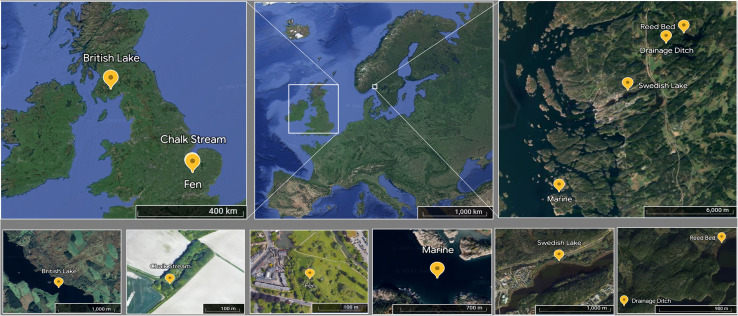
Location of each sampling site in a map of Europe. Yellow marks indicate each sampling locations at different spatial resolutions.

Samples were collected in Sweden and the UK (Fig. 1) to compare primarily the effects of shorter and longer freezing times, respectively. On 18 May 2022, surface water (0.5 m depth) was collected from three Swedish sites draining predominantly forested land: two lakes, Strömsvattnet (sampled from open water) and Ned Färingen (sampled in a reed bed), and a small drainage ditch. A coastal marine sample was obtained from Tjärno Marine Station located on the west coast of Sweden on 19 May 2022, off the end of a nearby jetty. We hereafter refer to these sites as Swedish lake (Strömsvattnet), reed bed (Ned Färingen), drainage ditch, and marine (Tjärno Marine Station). During July 2020, we collected samples from three sites in the UK: the River Mel (sampled on 17 July 2020), Coe Fen (sampled on 17 July 2020), and Loch Ken (sampled on 23 July 2020). The River Mel is a small, clear-water chalk stream located near the city of Cambridge. Coe Fen is a wetland inside the city of Cambridge that dries during the summer and is refilled during winter and after rainfall events. Loch Ken is a brown-water Scottish loch surrounded by forest plantations and agriculture in an area with relatively low population density. We hereafter refer to these sites as chalk stream (River Mel), fen (Coe Fen) and British lake (Scottish loch). All the UK samples were collected from surface waters (0.5 m depth) near the centre of the sampling site.

At each site we filtered six replicates into pre-combusted (4 h at 550 °C) glassware. In Sweden, we passed the water through pre-combusted 25 mm GF/F grade filters (Whatman, UK) loaded into a Swinnex syringe capsule (MilliporeSigma, USA), which is a standard protocol for DOM analyses of marine and freshwater samples.^6^ In the UK, we used pre-assembled 25 mm syringe filters with 0.2 μm cellulose acetate membranes (Sartorius, UK). Pre-assembled filters can be more practical in citizen science or sampling networks involving many different field teams, especially in remote field conditions where there may be limited access to laboratory facilities for pre-combustion of filter papers. At each site in Sweden and the UK, a new filter was used for each replicate sample, and the first 30 mL filtrate was discarded. Three replicates were acidified to a pH of 2 using 37% HCl, and prepared for SPE, and three replicates were transferred to a −20 °C freezer where they were stored for 21 (Sweden) or 95 days (UK). After the freezing period, samples were thawed in a fridge (*ca.* 4 °C) for 24 hours (Sweden) or at room temperature for 3 hours (UK), acidified to a pH of 2 using 37% HCl, and prepared for SPE. During thawing, one vial broke from the River Mel, and the water was transferred to a clean glass beaker for analysis. At each site, we collected an additional sample as described above into a pre-combusted 40 mL glass vial, which was kept in the dark at *ca.* 4 °C for up to one week prior to quantification of DOC concentration.

### 2.3 Solid phase extraction

Agilent PPL cartridges (100 mg styrene-divinylbenzene)^6^ were pre-conditioned using a 3 mL MeOH flush followed by a 4 hour MeOH soak and a 3 mL acidified Milli-Q™ (0.1% HCl) flush. Samples were loaded into 60 mL syringes and left to gravity feed the cartridges. A second 3 mL acidified Milli-Q™ flush was used to remove any salts before eluting the cartridges with 2 mL MeOH, approximately 1.8 mL of which was recovered into pre-combusted 4 mL amber vials. The vials were stored upright at −20 °C until analysis. On each occasion, SPE was also performed on a Milli-Q™ blank. The volume of samples extracted is presented in ESI Table 1.†

### 2.4 Mass spectrometry analysis

For each replicate in Sweden, 1.2 mL eluate was pipetted into a Milli-Q™ rinsed Eppendorf™ tube and dried in a Speedvac for 3 hours at 30 °C. Dry samples were then re-dissolved in 100 μL ACN + 0.1% FA, and brought back into solution by vortexing then sonicating for 5 minutes. The supernatant (80 μL) was then pipetted into a pre-combusted autosampler vial (1.5 mL with 300 μL inserts). 10 μL of sample was injected into the LC-MS for freshwater samples and 30 μL for marine samples.

For the UK replicates, the amount of eluate to be dried was determined based on an expected 60% extraction efficiency and with a target concentration of 100 ppm. Dried eluates were redissolved in 120 μL ACN + 0.1% FA containing internal standards of capsaicin (100 μL of 1000 ppm stock), adenosine-5-monophosphate (10 μL of ppm 1000 stock), raffinose (100 μL of 1000 ppm stock), carbenoxolone disodium (10 μL of 1000 ppm stock), and glycyrrhizic acid (10 μL of 1000 ppm stock), and the supernatant (100 μL) was transferred to an autosampler vial by Hamilton syringe which was washed between samples with methanol. 80 μL of each sample was injected into the LC-MS.

The MS (Orbitrap LTQ Velos, Thermo Fisher, Germany) was optimised in negative ion mode using direct infusion of a 20 ppm SRNOM solution in 50% methanol, tuning to maximise the intensity of the ion at 369.11911. Analytes were separated by reversed phase chromatography with a Kinetix Polar C18 column (100 × 2.1 mm, 2.6 μm, Phenomenex, Torrance, USA) and two mobile phases (A and B). Mobile phase A was 0.1% FA in LCMS grade H_2_O (*i.e.* 100 mL H_2_O + 100 μL FA). Mobile phase B was 0.1% FA in 80 : 20 (v/v) LCMS grade ACN : water. A second pump provided a counter gradient post-column (Han *et al.*, 2021), balancing the solvent composition to a constant 40% ACN. The mobile phases in the second pump were spiked with hippuric and fusidic acid (10 μL of 1000 ppm stock each per 100 mL) for the Swedish samples and hippuric, fusidic and cyclohexyl succinic acid (10 μL of 1000 ppm stock each per 100 mL) for the UK samples. The spiked compounds are expected to be absent in DOM samples and allow continuous calibration of mass at various *m*/*z* values.^22^ Compounds were separated over a 10 minute gradient: 0% B for 0.5 minutes, up to 100% B at 6 minutes, isocratic till 6.5 minutes, down to 0% B at 7 minutes, 3 minute equilibration at 0.22 mL min^−1^. After mixing with the counter gradient mobile phase, 10% of the total flow was diverted to the electrospray ionisation (ESI) source for analysis by UHRMS. The ESI was set to −3 kV, 100 °C, and the Orbitrap was set to collect data at a resolution setting of 60 000 (the second highest setting), to increase the total number of transients collected. The data were exported as a single integrated mass list.

### 2.5 DOC concentration

DOC samples were analysed on a Shimadzu TOC-V (Sweden) or TOC-L (UK) analyser (Shimadzu, Japan) and quantified as non-purgeable organic carbon (NPOC). Measurements were made in triplicate. For Swedish samples, pure water (Milli-Q) blanks and an ethylenediaminetetraacetic acid (EDTA, 8 mg C per L) standard were included throughout the analysis run. For UK samples, deionised (DI) water blanks and a 10 mg C per L potassium hydrogen phthalate standard were used throughout for QA/QC. For both studies, the measured values were within 5% of the expected values.

Before sampling, we also determined the concentration of water required to rinse the cellulose acetate filters used in the UK so that they did not release any DOC. Briefly, we flushed DI water in increments of 10 mL from 0 to 50 mL through a cellulose acetate syringe filter. Then, we passed an additional 25 mL of DI water through the filter and collected the filtrate. We measured DOC concentration in the filtrate and in the DI water. Passing 20 mL of DI water through the filter was enough to reduce the DOC to the original concentration of the DI water (mean ± standard error: filtered DI = −0.003 ± 0.052 mg C per L; original DI = 0.008 ± 0.031 mg C per L, *N* = 3).

### 2.6 Data analysis

A formula assignment routine was written in MATLAB (version R2021b, Mathworks, USA), which is available along with raw sample data in the ESI.† Formulas were assigned between masses 150–850 Da, with the following constraints: C 4–50, H 4–100, O 2–40, N 0–2, S 0–1, 13C 0–1 H/C 0.3–2.2, O/C 0–1, double bond equivalence-oxygen > −10. Noise was removed using the Kendrick mass defect slice method,^23^ and high molecular weight doubly charged interference peaks were removed from consideration before assignment.^22^ A mass error of 1 ppm and 1.75 ppm was allowed for the Swedish dataset and UK dataset respectively, after evaluating assignment errors after calibration (Fig. SI1 and 2†). All peaks that were not assigned a molecular formula were removed from the dataset. Remaining peak intensities were normalised to sum 1 × 10^6^ for each sample, and normalised intensities were used both for averaging (for comparison before and after freezing), and as the basis of Bray–Curtis dissimilarity testing.

We tested the effect of freezing on the number of peaks, the intensity weighted average O/C (O/C_wa_), H/C (H/C_wa_), and mass-to-charge (*m*/*z*_wa_) ratios using linear models. The linear models were fitted to each metric using the function *lm* in R 4.1.1, with predictor of site and treatment and their interaction, *i.e.* to test if freezing effects varied with DOM composition. Furthermore, for each site, individual peak intensities before and after freezing were compared using paired *t*-tests. To compare overall composition of samples, the Bray–Curtis dissimilarity between each sample was computed and used for visualising the variability between treatments with a principal coordinate analysis (PCoA).

## 3. Results

### 3.1 Short-term (21 day) storage of Swedish fresh and marine waters

**Fig. 2:**
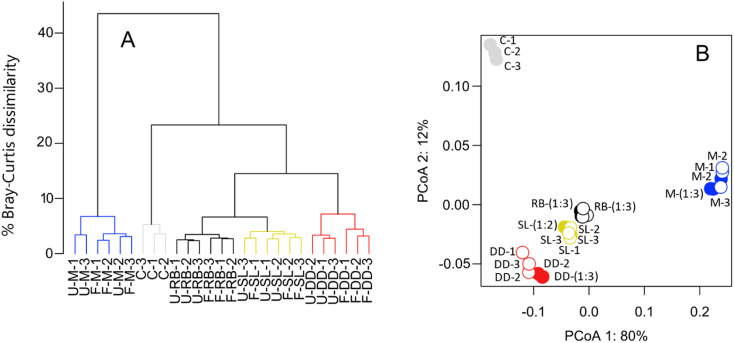
Freezing has negligible change on DOM composition after 21 days. Panel (A) shows a hierarchical cluster analysis of each sample before (U) and after (F) freezing. Differences were estimated between samples using the Bray–Curtis dissimilarity index. Panel (B) shows a principal coordinate analysis (PCoA) of sample distances based on Bray–Curtis dissimilarity of mass-intensity lists. Numbers along each axis correspond to the percentage of variation that they explain. Filled and open symbols indicate unfrozen and frozen samples, respectively. Colours correspond to each site shown in panel (A). RB = reed bed, SL = Swedish lake, DD = drainage ditch, M = marine, C = control (SRNOM).

Freezing samples from the Swedish sites for three weeks did not have a statistically significant effect on any of the peak metrics under investigation (Table 1). The mean and standard deviation of the intensity weighted peak metrics among replicate samples within frozen or unfrozen (*i.e.*, processed immediately after sampling) treatments were more similar than between treatments (frozen minus unfrozen samples, Table 1). Furthermore, freezing samples did not increase the dissimilarity between replicates, irrespective of the sites considered (Fig. 2 and Table 2). The effect size, measured as the difference in Bray–Curtis dissimilarities between unfrozen samples only *versus* between frozen and unfrozen samples, ranged from an average of 0.0 to 2.7% (Table 2).

**Table 1:** High resolution mass spectrometry peak metrics showing average number of peaks (peaks) and mean intensity weighted average of oxygen to carbon (O/C_wa_), hydrogen to carbon (H/C_wa_) and mass to charge (*m*/*z*_wa_) ratios. *N* = 3 for each sample set (unfrozen or frozen), and *N* = 9 for the comparison between frozen and unfrozen samples. SD = standard deviation. There was no statistically significant difference between frozen and unfrozen samples (*p* > 0.05)

Sample	Peak (SD)	O/C_wa_ (SD)	H/C_wa_ (SD)	*m*/*z*_wa_ (SD)
SRNOM	4103 (90)	0.58 (0.01)	1.03 (0.01)	417.58 (0.14)
Marine (unfrozen)	4738 (30)	0.50 (0.00)	1.22 (0.00)	376.03 (0.70)
Marine (frozen)	4889 (238)	0.51 (0.00)	1.23 (0.00)	374.55 (0.86)
Marine (frozen–unfrozen)	106 (208)	0.00 (0.00)	0.00 (0.02)	−1.5 (0.97)
Drainage ditch (unfrozen)	4774 (15)	0.52 (0.00)	1.07 (0.00)	415.24 (0.67)
Drainage ditch (frozen)	4747 (91)	0.53 (0.00)	1.06 (0.01)	416.82 (2.32)
Drainage ditch (frozen–unfrozen)	−27 (80)	0.01 (0.01)	−0.02 (0.01)	1.57 (2.09)
Reed bed (unfrozen)	5119 (25)	0.53 (0.00)	1.10 (0.00)	401.53 (0.46)
Reed bed (frozen)	5036 (29)	0.52 (0.00)	1.11 (0.00)	400.75 (0.13)
Reed bed (frozen–unfrozen)	−83 (33)	−0.00 (0.00)	0.00 (0.00)	−0.77 (0.41)
Swedish lake (unfrozen)	5057 (55)	0.53 (0.00)	1.09 (0.00)	402.01 (0.69)
Swedish lake (frozen)	5012 (40)	0.53 (0.00)	1.09 (0.00)	401.59 (0.30)
Swedish lake (frozen–unfrozen)	−45 (59)	0.00 (0.01)	0.00 (0.01)	−0.43 (0.65)

**Table 2:** DOM composition is similar among replicates of a site. We calculated the mean Bray–Curtis dissimilarity (BCD) for all pairwise comparisons of replicates both before (U) and after freezing (F) and between the freezing treatments for each sampling site. The effect size is the difference between the mean BCD comparing unfrozen samples with unfrozen samples and the mean BCD comparing unfrozen samples with frozen samples. SD = standard deviation

Site	Unfrozen *vs.* unfrozen (SD)	Frozen *vs.* frozen (SD)	Unfrozen *vs.* frozen (SD)	Effect size (BCD_F_–BCD_U_)	[DOC] mg C per L
Marine	4.2 (0.9)	3.0 (0.6)	5.1 (1.4)	0.9	2.8
Drainage ditch	3.1 (0.3)	3.8 (0.6)	5.8 (0.9)	2.7	18.6
Reed bed	2.4 (0.1)	3.8 (0.6)	3.1 (0.2)	0.7	90.5
Swedish lake	3.4 (0.7)	3.1 (0.4)	3.4 (0.4)	0.0	16.1

**Fig. 3:**
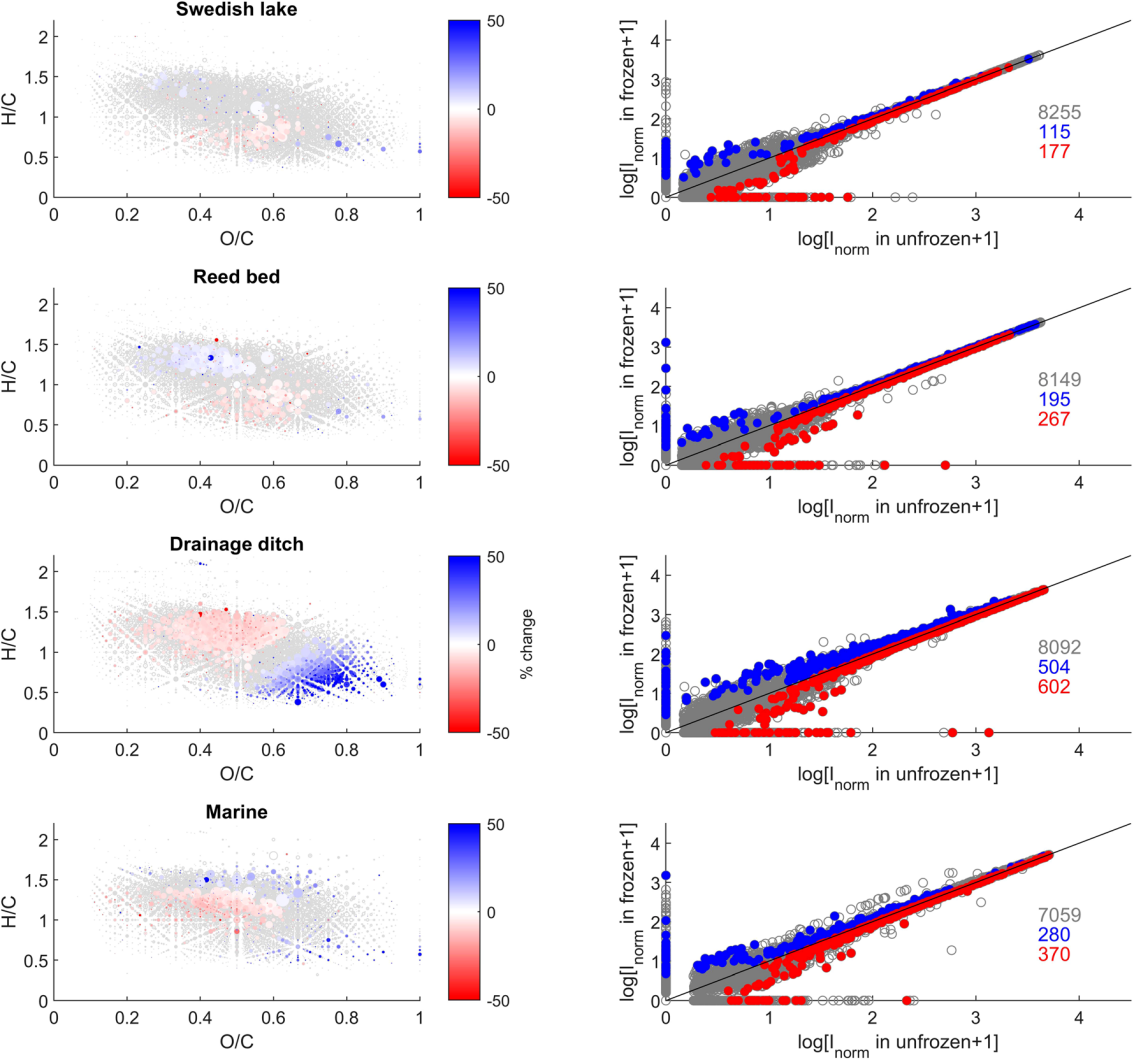
van Krevelen (VK) diagrams (Left) and intensity comparisons (Right) after freezing samples for 21 days. Intensities are shown as size of point for VK diagrams and as logarithms in the right panel. All peaks are shown in grey (open circles), and those with a statistically significant relative decrease and increase after freezing are superimposed in red and blue (filled circles) as a gradient of % change, respectively. In the right panel, the logarithm of average intensity (*I*_norm_) of unfrozen and frozen samples are shown on *x*- and *y*-axes, respectively. Unchanged peaks are shown as grey circles, and red/blue colours signify decreased/increased peaks, respectively, corresponding to the van Krevelen diagram. The numbers in the mass spectra are the number of peaks in each group.

We observed statistically significant changes in the intensity of some individual peaks (Fig. 3). We found a total of 8255, 8149, 8092, and 7059 peaks were present in at least one of the replicates from the Swedish lake, reed bed, drainage ditch, and marine sites, respectively (Fig. 3). Of those peaks, 3, 6, 14, and 9% (with a sum intensity respectively corresponding to, 8, 7, 6, and 12% of the total sum intensity of each sample) were significantly (paired *t*-test, *p* < 0.05) affected by freezing in the Swedish lake, reed bed, drainage ditch, and marine sites, respectively. Molecular formulae exhibiting altered intensity varied depending on the sampling site. In the drainage ditch and marine sample, compounds with a H/C > 1 and O/C of 0.2–0.6, often assigned to carboxyl-rich alicyclic molecules (CRAMs), decreased in relative intensity after freezing (Fig. 3). In the drainage ditch, molecular formulae with a H/C < 1 and O/C > 0.4, often associated with tannins, decreased in relative intensity. Other sites had very minor changes without clear patterns in van Krevelen space (Fig. 3).

### 3.2 Long-term (95 days) storage of UK freshwaters

**Fig. 4:**
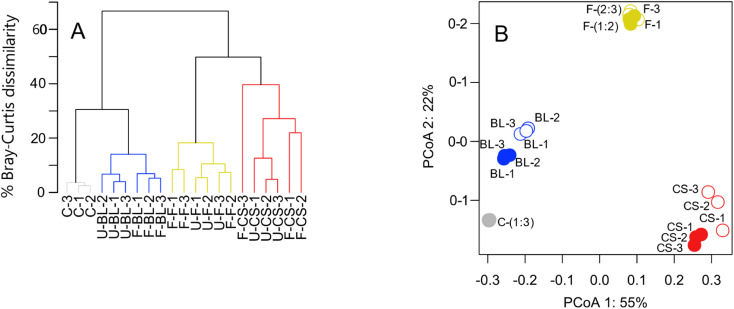
Freezing has little effect on DOM composition after 95 days except at lower DOC concentrations. Panel (A) shows a hierarchical cluster analysis of each sample before (U) and after (F) freezing. A control (C) of SRNOM measured from a single sample was included without freezing to measure expected technical variation between replicates due to the SPE extraction and UHRMS process. Differences were estimated between samples using the Bray–Curtis dissimilarity index. Panel (B) shows a principal coordinate analysis (PCoA) of sample distances based on Bray–Curtis dissimilarity of mass-intensity lists. Numbers along each axis correspond to the percentage of variation that they explain. Filled and open symbols indicate unfrozen and frozen samples, respectively. Colours correspond to each site shown in panel (A). BL = British lake, F = fen, CS = chalk stream.

Freezing samples for 95 days had a statistically significant effect on intensity weighted peak metrics for the chalk stream and British lake (Table 3). For the chalk stream, the number of peaks detected and the *m*/*z*_wa_ ratio decreased by 45% and 6%, on average, respectively (Table 3). For the British lake, the O/C_wa_, and the *m*/*z*_wa_ ratio decreased by 2%, and 4%, on average, respectively, whereas the H/C_wa_ increased by 7%, on average (Table 3). The Bray–Curtis dissimilarity between replicates were smaller before compared with after freezing samples for the chalk stream and British lake (Fig. 4). The difference in peak intensity between replicates was exacerbated by one replicate for the chalk stream, for which the vial broke during processing, and that was 30% dissimilar to the other replicates (F-CS-3 in Fig. 4). The effect size, measured as the difference in Bray–Curtis dissimilarities between unfrozen samples and the Bray–Curtis dissimilarities between frozen and unfrozen samples, ranged from an average of 5.0% to 18.2% (Table 4).

**Table 3:** High resolution mass spectrometry peak metrics showing number of peaks (peaks) and the intensity weighted average of oxygen to carbon (O/C_wa_), hydrogen to carbon (H/C_wa_) and mass to charge (*m*/*z*_wa_). Bolded numbers indicate a statistically significant difference (*p* < 0.05) after freezing. *N* = 3 for each sample set (unfrozen or frozen), and *N* = 9 for the comparison between frozen and unfrozen sample. SD = standard deviation

Sample	Peak (SD)	O/C_wa_ (SD)	H/C_wa_ (SD)	*m*/*z*_wa_ (SD)
SRNOM	3828 (97)	0.62 (0.00)	1.01 (0.00)	428.6 (1.1)
Chalk stream (unfrozen)	1701 (14)	0.50 (0.01)	1.22 (0.03)	417.31 (1.62)
Chalk stream (frozen)	941 (114)	0.49 (0.02)	1.29 (0.10)	391.99 (10.60)
Chalk stream (frozen–unfrozen)	**−760 (127)**	−0.01 (0.02)	0.06 (0.11)	**−25.32 (12.10)**
British lake (unfrozen)	2432 (252)	0.55 (0.00)	1.04 (0.00)	424.96 (1.05)
British lake (frozen)	2101 (114)	0.54 (0.01)	1.11 (0.01)	408.05 (3.66)
British lake (frozen–unfrozen)	−331 (142)	**−0.01 (0.00)**	**0.07 (0.01)**	**−16.90 (4.46)**
Fen (unfrozen)	1657 (217)	0.49 (0.00)	1.25 (0.00)	353.20 (1.68)
Fen (frozen)	1579 (91)	0.49 (0.01)	1.28 (0.03)	352.10 (6.68)
Fen (frozen–unfrozen)	−78 (168)	−0.01 (0.00)	0.03 (0.03)	−1.10 (5.91)

**Table 4:** Bray–Curtis dissimilarity between unfrozen or frozen replicates, and among all unfrozen (U) and frozen (F) replicates. Values are means ± standard deviation across all potential pairwise comparisons. The effect size represents the difference between the mean BCD comparing unfrozen samples with unfrozen samples and the mean BCD comparing unfrozen samples with frozen samples. SD = standard deviation

Site	Unfrozen *vs.* unfrozen (SD)	Frozen *vs.* frozen (SD)	Unfrozen *vs.* frozen (SD)	Effect size (BCD_F_–BCD_U_)	[DOC] mg C per L
SRNOM	3.2 (0.6)	—	—	—	20.0
Chalk stream	9.4 (3.6)	30.0 (7.0)	27.6 (8.0)	18.2	1.5
British lake	5.6 (1.3)	6.0 (0.7)	11.7 (1.4)	6.1	7.6
Fen	8.2 (2.3)	10.6 (2.3)	13.2 (3.9)	5.0	4.1

**Fig. 5:**
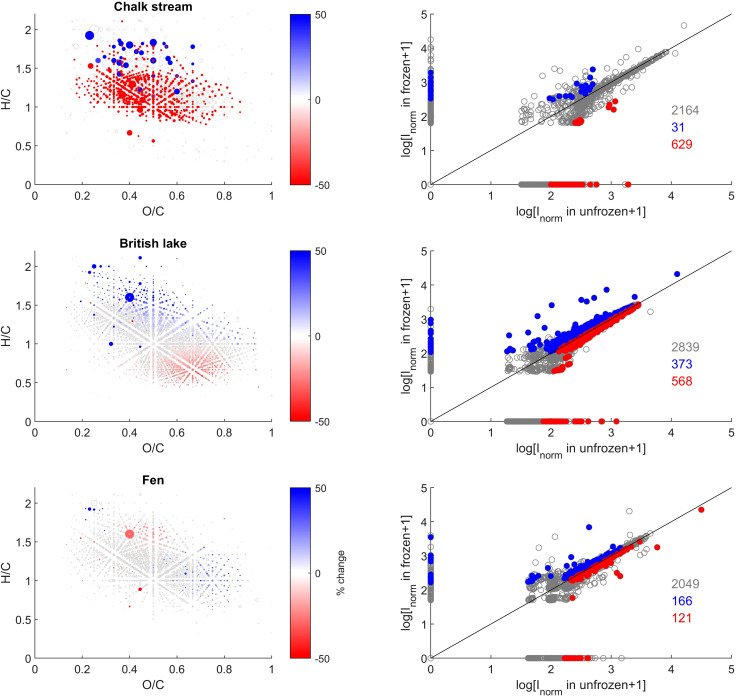
van Krevelen (VK) diagrams (Left) and intensity comparisons (Right) after freezing samples for 95 days. Intensities are shown as size of point for VK diagrams and as logarithms in the right panel. All peaks are shown in grey (open circles), and those with a statistically significant relative decrease and increase after freezing are superimposed in red and blue (filled circles) as a gradient of % change, respectively. In the right panel, the logarithm of average intensity (*I*_norm_) of unfrozen and frozen samples are shown on *x*- and *y*-axes, respectively. Unchanged peaks are shown as grey circles, and red/blue colours signify decreased/increased peaks, respectively, corresponding to the van Krevelen diagram. The numbers in the mass spectra are the number of peaks in each group.

More pronounced changes were found at the level of individual peak abundances in the second study compared with the first (Fig. 3 and 5). We found a total of 2164, 2839, and 2049 peaks were present in at least one of the replicates from the chalk stream, British lake, and fen sites, respectively (Fig. 3). Of those peaks 28, 29% and 11% (respectively 11, 45, and 13% of the intensity) were significantly (paired *t*-test, *p* < 0.05) affected by freezing for chalk stream, British lake, and the fen sites respectively. For the chalk stream, most changes occurred for compounds related to CRAMs (H/C > 1 and O/C of 0.2–0.6), with these mostly decreasing in relative intensity (Fig. 5) like in the Swedish drainage ditch and marine sample (Fig. 2). For the British lake, compounds related to aliphatics (H/C > 1.5 and O/C of 0.2–0.6) increased in relative intensity, whereas compounds associated with polyphenols (H/C 0.5–1.0 and O/C > 0.6) decreased in relative intensity (Fig. 5). For the fen, few clear patterns emerged in van Krevelen space (Fig. 5).

### 3.3 Technical reproducibility

In our dataset, the SRNOM peak metrics varied the least on each occasion, as these were analytical replicates from a single vial. On average, we found more peaks, a higher H/C_wa_ ratio, a lower O/C_wa_ ratio, and a lower *m*/*z*_wa_ in the SRNOM processed with the Swedish sites (Tables 1 and 5) than the SRNOM processed with the UK sites (Tables 3 and 5). However, the differences in peak metrics between the SRNOM remained small and did not affect the conclusion of this study. The intensity weighted average metrics for the SRNOM standard were also compared with recent data from an international ring trial.^5^ Because the current data were obtained by liquid chromatography separation with counter gradient, which increases the detection of compounds with a high O/C ratio,^24^ and not by direct infusion as used in the ring trial, some differences were expected. Compared with the direct infusion of the ring trial, we found that high O/C and low H/C peaks were slightly over-represented in our analysis, along with higher mass compounds (especially for the UK sites), but that our instrument and method remained suitable for analysing DOM (Table 5).

**Table 5:** Average and standard deviation of metrics for the ‘Commonly Detected Peaks’ in Suwannee River natural organic matter compared with mean and standard deviation of all labs in the recent interlaboratory comparison.^5^ Data from this study were from a chromatographic separation, and the interlaboratory comparison used direct infusion

	Case 1 (Sweden)	Case 2 (UK)	Interlaboratory study
O/C_wa_	0.58 ± 0.00	0.62 ± 0.00	0.57 ± 0.02
H/C_wa_	1.05 ± 0.00	1.02 ± 0.00	1.05 ± 0.02
*m*/*z*_wa_	401.4 ± 0.2	404.07 ± 0.22	405 ± 17

## 4. Discussion

### 4.1 Effect of freezing on DOM composition

In this study, we have demonstrated that freezing samples does not have major consequences on overall peak metrics (H/C, O/C, *m*/*z*, or peak number) or on the intensity of individual peaks in northern temperate waters. Specifically, freezing samples had smaller effects on DOM composition than reports of other sample preparation techniques routinely used in DOM sample analysis by mass spectrometry. Our results showed that compositional changes induced by freezing were usually much less than 10% (Tables 2 and 4). Therefore, like SPE^25^ and choice of ionisation technique,^26,27^ sample freezing does influence sample composition. However, the effects of freezing were lower than those of SPE, which has been shown to increase the difference between technical replicates by *ca.* 15% for samples processed with a styrene-divinylbenzene polymer (PPL) sorbent.^15^ Furthermore, more than 40% of the carbon is usually lost during the SPE process, irrespective of freezing, indicating that a large fraction of the sample is not recovered.^25,28^ Ionisation techniques are also hugely important in dictating which peaks are observed,^26,29^ and electrospray source geometry and settings may be one of the main reasons for different results between laboratories.^5^ A recent estimate indicates that only 33% of the standard Suwannee River fulvic acid is efficiently ionised by ESI in negative mode.^27^ Overall, the combination of SPE and ESI provides the analyst with a very specific, but nevertheless important analytical window through which the sample is viewed. The addition of the minor sample freeze–thaw effect on top of these major biases should be considered a ‘necessary evil’ in cases where, for logistical or archiving reasons, the sample needs to be frozen. Furthermore, unlike for SPE or sample ionisation, it is possible to both quantify and characterise the effects of freezing, rendering data interpretation more manageable.

The effects of freezing are highly consistent and reproducible. Out of 21 frozen samples, only one replicate was clearly different from the other replicates. That replicate (F-CS-3, Fig. 4) was broken during thawing, and it is likely that unnoticed contamination occurred during transfer to new glassware. The potential for contamination emphasises the need for sample replication, which is often not undertaken in UHRMS DOM studies.^5^ Our results suggest very high reproducibility of the results between replicates both before and after freezing, and in most cases between unfrozen and frozen samples. In the few cases where there was a statistically significant change after freezing (*e.g.* for the chalk stream), freezing primarily decreased the peak number and average mass, and not the O/C or H/C metrics (Tables 1 and 3).

Compositional changes underlying shifts in peak intensity reflected the sampling environment. The reed bed (Sweden) and British lake (UK) exhibited an increase in aliphatic and CRAM formulas, while more aromatic formulas decreased (Fig. 3 and 5). Conversely, the more terrestrial drainage ditch (Sweden) and fen (UK) sites, exhibited an increase in tannin-like compounds and decrease in more aliphatic compounds, essentially the opposite effect. The marine site (Sweden), Swedish lake (Sweden) and chalk stream (UK) had no consistent patterns in van Krevelen space related to freezing (Fig. 3 and 5), but as stated, peak numbers decreased considerably for the chalk stream (Table 3 and Fig. 5). These effects suggest that the intrinsic properties of compounds, such as 3D structures and intermolecular bonds, and compound chemistry related to site (bio)geochemistry does lead to freezing effects, and that these effects are consistent and reproducible between biogeochemically similar sites.

Our study may also have implications for freezing of methanol extracts after SPE. This practice is widespread but may similarly affect DOM composition. It has previously been determined that, at room temperature, DOM carboxylic acid groups can form methyl esters with solvent methanol.^30^ This reaction is much slower at −20 °C, but presumably not halted entirely over years of storage. Extracts could be stored after drying,^31^ but this practice is uncommon and the effects of drying remain unknown. Furthermore, drying extracts or samples under field conditions is often impractical due to equipment requirements. Future efforts should attempt to determine the effects of long-term storage at −20 °C on methanol extracts if the need to re-measure samples becomes relevant, particularly if this is considered a better alternative to freezing of site water.

### 4.2 Comparison between the two case studies

We observed a smaller effect of freezing after 21 days than after 95 days. Besides the freezing time, the type of filter, the volume of sample extracted, the amount of carbon injected into the HPLC-Orbitrap, and the sampling sites differed between the two experiments. Because both types of filters were flushed and no leaching of carbon was observed for the cellulose acetate filters, it is unlikely that the type of filter contributed to the differences between the two experiments. Passing more water through the cartridge only increased the number of peaks detected in the UK sites (Table S1†) and is therefore unlikely to explain the observed effects of freezing between the Swedish and UK studies (Tables 2 and 4). However, future studies should aim to keep extraction volumes as consistent as possible to avoid potential differences during analysis. Sites in Sweden and the UK differed in their concentrations of inorganic ions, which might interfere with the extraction efficiency of DOM. Furthermore, two of the three UK sites had a higher H/C ratio than three of the four Swedish sites (Tables 1 and 3), indicating a broad difference in DOM chemistry between the two sets. Therefore, our results suggest that the larger effect of freezing in the UK sites is likely caused by the longer freezing time, differences in water chemistry, or differences in extraction volumes. Further work would be required to fully deconvolute site (*e.g.* salt content, DOC concentration) and storage time effects.

## 5. Conclusions

In this study, we showed that freezing can change DOM composition, but that any changes are relatively small (typically < 10%) and can be both quantified and characterised. Importantly, freezing never impeded distinction of DOM from different study sites. A good practice for future UHRMS studies of frozen DOM would be to quantify and characterise biases with a set of contrasting reference materials, in addition to ensuring sample replication. Furthermore, we found that the duration of freezing between 21 and 96 days did not have strong effects on DOM composition. These results suggest that samples may remain representative of their original composition even after many more months, thereby offering the potential to analyse long-term archives. Nonetheless, further studies evaluating the effects of freezing over very long time scales (*e.g.* years) would be beneficial. Our results also raise new questions about processing frozen methanol extracts, as is routinely done^4,6^ during UHRMS. Some of the effects we observed in water samples might also occur in methanol extracts, in addition to methylation of carboxylic acids to form methyl esters. Overall, we conclude that freezing samples can produce a small but measurable bias that may be acceptable for large-scale and/or remote environmental studies.

## Conflicts of interest

There are no conflicts to declare.

## Supplementary Material

RA-013-D3RA01349A-s001
