# Supplementary material for: Assessment of sample freezing as a preservation technique for analysing the molecular composition of dissolved organic matter in aquatic systems
Source: RSC Adv. 2023 Aug 16;13(35):24594–603. doi: 10.1039/d3ra01349a (PMC10427896; doi:10.1039/d3ra01349a)
Supplement: RA-013-D3RA01349A-s001 [file RA-013-D3RA01349A-s001.pdf]

Electronic Supplementary Material (ESI) for RSC Advances.
This journal is © The Royal Society of Chemistry 2023

Supplementary information for: Assessment of sample freezing as a preservation technique for analysing the molecular composition of dissolved organic matter in aquatic systems

Jeremy A. Fonvielle[1□], Stacey L. Felgate[2□], Andrew J. Tanentzap[1,3] and Jeffrey A. Hawkes[2]*

[1] Ecosystems and Global Change Group, Department of Plant Sciences, University of Cambridge, Cambridge, United Kingdom CB2 3EA.

[2] Department of Chemistry - BMC, Husargatan 3, Uppsala University, Uppsala, Sweden 752 37.

[3] Ecosystems and Global Change Group, School of the Environment, Trent University, Peterborough, Canada K9L 0G2.

* Corresponding author: J. Hawkes (jeffrey.hawkes@kemi.uu.se)

[□] J. A. Fonvielle and S. L. Felgate share first authorship of this paper.

Contents:

Supplementary figure 1&2: Error analysis for formula assignments in both cases

Supplementary table 1: sample processing information and mass spectrometry metrics

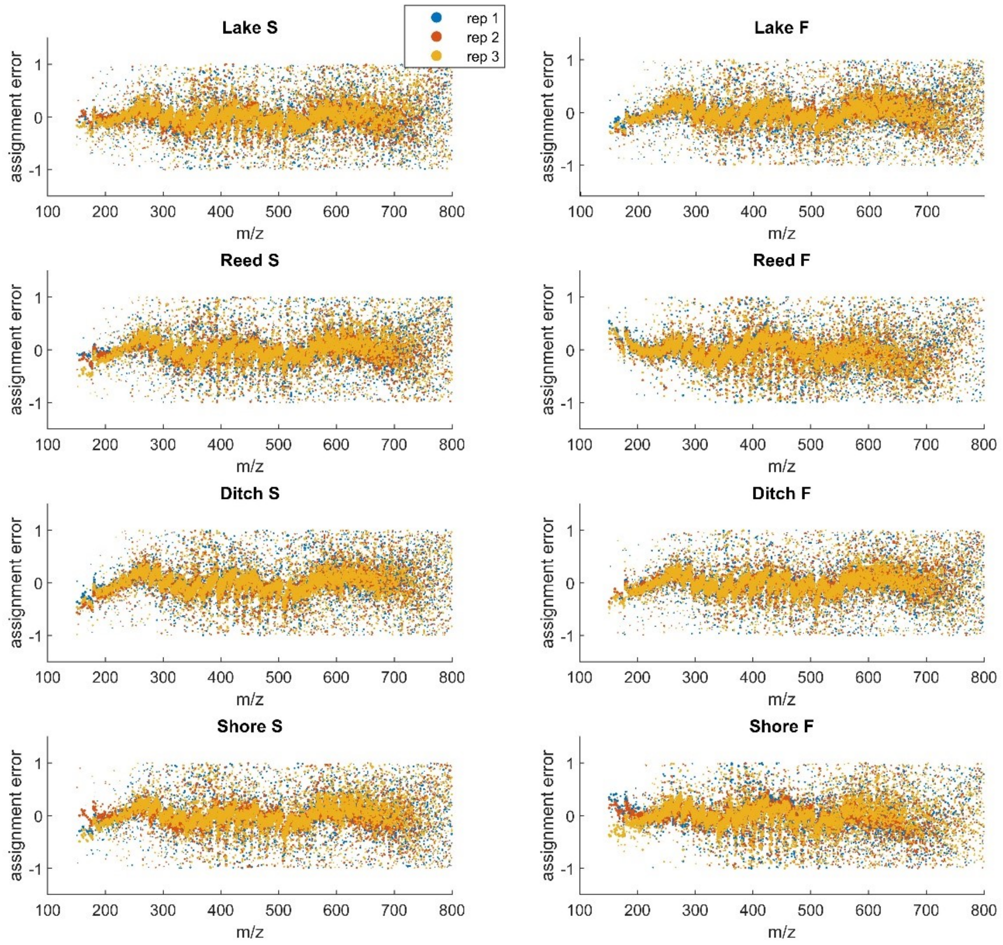


**Figure SI1:** Error analysis for the Swedish systems. Assignment error in ppm plotted vs mass to charge ratio, with each of three replicates overlaid. Point size is shown as logarithm of intensity to emphasise the most intense peaks. See methods for further information on molecular assignment errors.

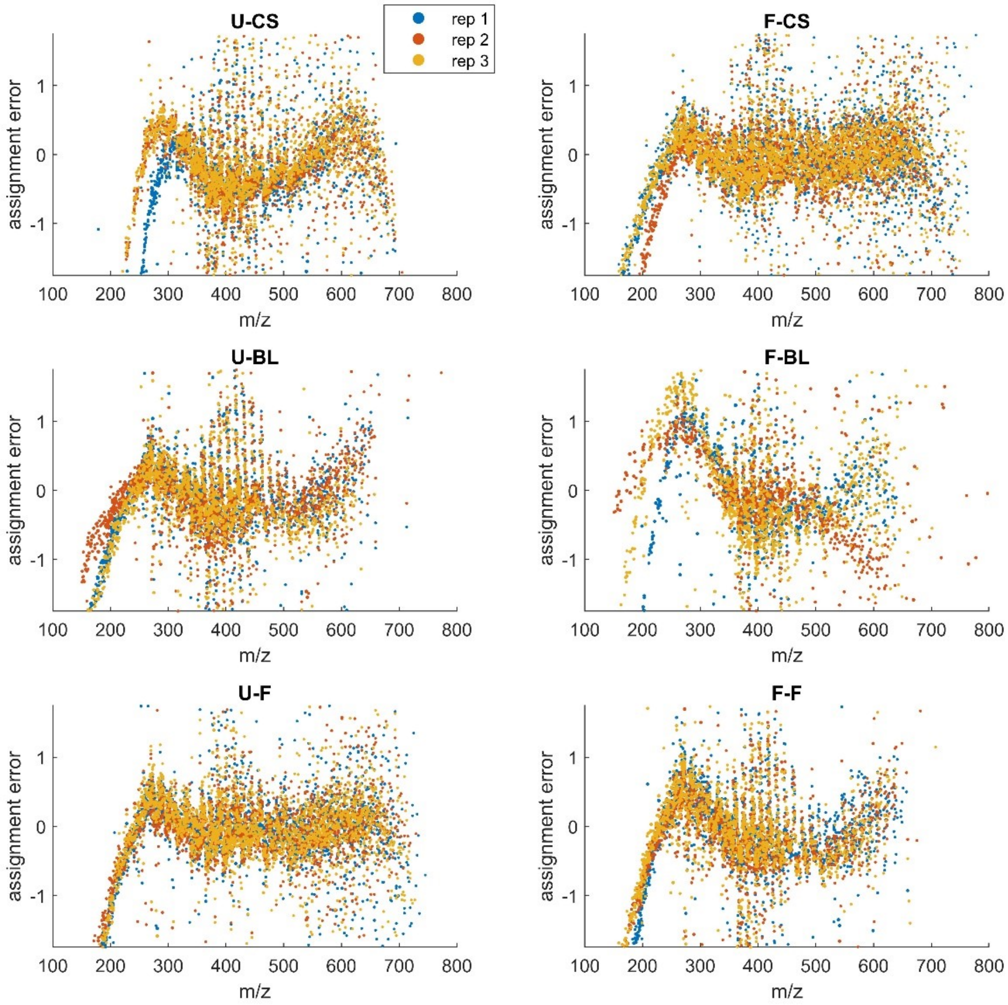


**Figure SI2:** Error analysis for the UK systems. Assignment error in ppm plotted vs mass to charge ratio, with each of three replicates overlaid. Point size is shown as logarithm of intensity to emphasise the most intense peaks. See methods for further information on molecular assignment errors.

**Table S1:** *Sample processing information (carbon injected and Volume of sample extracted) alongside high-resolution mass spectrometry peak metrics showing number of peaks (Peaks) and the intensity weighted average of oxygen to carbon ($O/C_{wa}$), hydrogen to carbon ($H/C_{wa}$) and mass to charge ($m/z_{wa}$) for each sample.*

| Sample | Country | Frozen | Sample code | Peaks | Total current | $O/C_{wa}$ | $H/C_{wa}$ | $m/z_{wa}$ | millilitres Extracted |
|---|---|---|---|---|---|---|---|---|---|
| **Marine** | SW | N | U-M-1 | 2074 | 1.64E+06 | 0.5 | 1.25 | 361.91 | 54 |
| **Marine** | SW | N | U-M-2 | 2018 | 1.85E+06 | 0.5 | 1.26 | 362.52 | 54 |
| **Marine** | SW | N | U-M-3 | 2154 | 1.67E+06 | 0.48 | 1.28 | 364.33 | 54 |
| **Marine** | SW | Y | F-M-1 | 2142 | 2.61E+06 | 0.5 | 1.25 | 362.17 | 53 |
| **Marine** | SW | Y | F-M-2 | 2527 | 2.41E+06 | 0.51 | 1.26 | 359.78 | 53 |
| **Marine** | SW | Y | F-M-3 | 2292 | 2.66E+06 | 0.5 | 1.25 | 361.62 | 56 |
| **Swedish Lake** | SW | N | U-SL-1 | 3804 | 3.25E+06 | 0.52 | 1.12 | 404.35 | 54 |
| **Swedish Lake** | SW | N | U-SL-2 | 3466 | 3.10E+06 | 0.52 | 1.12 | 397.62 | 54 |
| **Swedish Lake** | SW | N | U-SL-3 | 3442 | 2.91E+06 | 0.51 | 1.13 | 401.71 | 54 |
| **Swedish Lake** | SW | Y | F-SL-1 | 3576 | 3.01E+06 | 0.51 | 1.12 | 402.6 | 53 |
| **Swedish Lake** | SW | Y | F-SL-2 | 3455 | 3.15E+06 | 0.52 | 1.12 | 402.65 | 55 |
| **Swedish Lake** | SW | Y | F-SL-3 | 3328 | 3.00E+06 | 0.52 | 1.12 | 397.15 | 53 |
| **Reed Bed** | SW | N | U-RB-1 | 3836 | 3.62E+06 | 0.52 | 1.13 | 403.75 | 54 |
| **Reed Bed** | SW | N | U-RB-2 | 3813 | 3.63E+06 | 0.51 | 1.13 | 404.14 | 54 |
| **Reed Bed** | SW | N | U-RB-3 | 4043 | 3.62E+06 | 0.51 | 1.13 | 403.47 | 54 |
| **Reed Bed** | SW | Y | F-RB-1 | 3532 | 3.28E+06 | 0.51 | 1.14 | 402.37 | 52 |
| **Reed Bed** | SW | Y | F-RB-2 | 3826 | 3.20E+06 | 0.51 | 1.13 | 403.57 | 53 |
| **Reed Bed** | SW | Y | F-RB-3 | 3904 | 3.45E+06 | 0.52 | 1.13 | 403.46 | 52 |
| **Drainage Ditch** | SW | N | U-DD-1 | 3561 | 3.05E+06 | 0.5 | 1.12 | 413.8 | 54 |
| **Drainage Ditch** | SW | N | U-DD-2 | 3680 | 3.22E+06 | 0.51 | 1.11 | 413.5 | 54 |
| **Drainage Ditch** | SW | N | U-DD-3 | 3682 | 3.05E+06 | 0.5 | 1.13 | 413.28 | 54 |
| **Drainage Ditch** | SW | Y | F-DD-1 | 3811 | 3.18E+06 | 0.52 | 1.09 | 417.21 | 53 |
| **Drainage Ditch** | SW | Y | F-DD-2 | 3851 | 3.21E+06 | 0.5 | 1.13 | 409.63 | 52 |
| **Drainage Ditch** | SW | Y | F-DD-3 | 3808 | 3.22E+06 | 0.51 | 1.11 | 413.43 | 52 |
| **Chalk Stream** | UK | N | U-CS-1 | 1699 | 2.15E+10 | 0.49 | 1.26 | 415.93 | 30 |
| **Chalk Stream** | UK | N | U-CS-2 | 1717 | 2.24E+10 | 0.51 | 1.22 | 416.89 | 30 |
| **Chalk Stream** | UK | N | U-CS-3 | 1689 | 2.25E+10 | 0.51 | 1.2 | 419.1 | 30 |
| **Chalk Stream** | UK | Y | F-CS-1 | 888 | 1.49E+10 | 0.5 | 1.28 | 396.79 | 19 |
| **Chalk Stream** | UK | Y | F-CS-2 | 863 | 1.33E+10 | 0.51 | 1.2 | 399.34 | 26 |
| **Chalk Stream** | UK | Y | F-CS-3 | 1073 | 2.38E+10 | 0.48 | 1.39 | 379.83 | 18 |
| **British Lake** | UK | N | U-BL-1 | 2592 | 2.84E+10 | 0.55 | 1.05 | 425.14 | 30 |
| **British Lake** | UK | N | U-BL-2 | 2142 | 2.63E+10 | 0.55 | 1.05 | 425.92 | 30 |
| **British Lake** | UK | N | U-BL-3 | 2564 | 3.29E+10 | 0.55 | 1.04 | 423.84 | 30 |
| **British Lake** | UK | Y | F-BL-1 | 2196 | 1.96E+10 | 0.55 | 1.1 | 410.64 | 23 |
| **British Lake** | UK | Y | F-BL-2 | 1974 | 2.16E+10 | 0.53 | 1.13 | 403.87 | 23 |
| **British Lake** | UK | Y | F-BL-3 | 2134 | 2.19E+10 | 0.54 | 1.11 | 409.67 | 26 |
| **Fen** | UK | N | U-F-1 | 1720 | 1.51E+10 | 0.49 | 1.25 | 352.96 | 30 |
| **Fen** | UK | N | U-F-2 | 1836 | 1.61E+10 | 0.49 | 1.25 | 354.99 | 30 |
| **Fen** | UK | N | U-F-3 | 1415 | 1.61E+10 | 0.49 | 1.25 | 351.64 | 30 |
| **Fen** | UK | Y | F-F-1 | 1671 | 2.02E+10 | 0.49 | 1.28 | 357.92 | 22 |
| **Fen** | UK | Y | F-F-2 | 1577 | 1.86E+10 | 0.5 | 1.24 | 353.56 | 22 |
| **Fen** | UK | Y | F-F-3 | 1489 | 2.09E+10 | 0.49 | 1.31 | 344.8 | 12 |
